# Identification of diagnostic biomarkers and therapeutic targets in peripheral immune landscape from coronary artery disease

**DOI:** 10.1186/s12967-022-03614-1

**Published:** 2022-09-05

**Authors:** Xiaoteng Feng, Yifan Zhang, Min Du, Sijin Li, Jie Ding, Jiarou Wang, Yiru Wang, Ping Liu

**Affiliations:** Department of Cardiology, Longhua Hospital, Shanghai University of Traditional Chinese Medicine, Shanghai, China

**Keywords:** Coronary atherosclerotic disease, Immune-related genes, Peripheral blood, Inflammation, Immune cells

## Abstract

**Background:**

Peripheral biomarkers are increasingly vital non-invasive methods for monitoring coronary artery disease (CAD) progression. Their superiority in early detection, prognosis evaluation and classified diagnosis is becoming irreplaceable. Nevertheless, they are still less explored. This study aimed to determine and validate the diagnostic and therapeutic values of differentially expressed immune-related genes (DE-IRGs) in CAD.

**Methods:**

We downloaded clinical information and RNA sequence data from the GEO database. We used R software, GO, KEGG and Cytoscape to analyze and visualize the data. A LASSO method was conducted to identify key genes for diagnostic model construction. The ssGSEA analysis was used to investigate the differential immune cell infiltration. Besides, we constructed CAD mouse model (low-density lipoprotein receptor deficient mice with high fat diet) to discover the correlation between the screened genes and severe CAD progress. We further uncovered the role of IL13RA1 might play in atherosclerosis.

**Results:**

A total of 762 differential genes were identified between the peripheral blood of 218 controls and 199 CAD patients, which were significantly associated with infection, immune response and neural activity. 58 DE-IRGs were obtained by overlapping the differentially expressed genes(DEGs) and immune-related genes downloaded from ImmpDb database. Through LASSO regression, CCR9, CER1, CSF2, IL13RA1, INSL5, MBL2, MMP9, MSR1, NTS, TNFRSF19, CXCL2, HTR3C, IL1A, and NR4A2 were distinguished as peripheral biomarkers of CAD with eligible diagnostic capabilities in the training set (AUC = 0.968) and test set (AUC = 0.859). The ssGSEA analysis showed that the peripheral immune cells had characteristic distribution in CAD and also close relationship with specific DE-IRGs. RT-qPCR test showed that CCR9, CSF2, IL13RA1, and NTS had a significant correlation with LDLR^−/−^ mice. IL13RA1 knocked down in RAW264.7 cell lines decreased SCARB1 and ox-LDL-stimulated CD36 mRNA expression, TGF-β, VEGF-C and α-SMA protein levels and increased the production of IL-6, with downregulation of JAK1/STAT3 signal pathway.

**Conclusions:**

We constructed a diagnostic model of advanced-stage CAD based on the screened 14 DE-IRGs. We verified 4 genes of them to have a strong correlation with CAD, and IL13RA1 might participate in the inflammation, fibrosis, and cholesterol efflux process of atherosclerosis by regulating JAK1/STAT3 pathway.

**Supplementary Information:**

The online version contains supplementary material available at 10.1186/s12967-022-03614-1.

## Introduction

Coronary artery disease (CAD) is still one of the leading causes of global death [1]. In 2017, CAD independently resulted in about 18 million deaths globally [2], and by 2030 as predicted, the number would reach 23.4 million [3]. Situations are becoming even worse under the context of the Covid-19 pandemic. Atherosclerotic lesions always result in progressive coronary stenosis, myocardial ischemia, necrosis and eventually the cumulative risk of acute cardiovascular syndrome (ACS). This process may last for decades. In the long course, patients usually experience peripheral blood immune component changes [4], which are related to the acute exacerbation, remission and stability of the disease. Therefore, they can also serve as biomarkers for disease monitoring or as targets for treatment. Effectively identification of them and subsequent mechanism exploration are critical for the secondary prevention of CAD.

Evidence accumulated in the past 20 years has shown that immune disorders played decisive roles in the initiation and development of the CAD pathology process [5]. Some immune changes have already existed before the pathological phenomenon occurs [6]. Previous bioinformatic studies had extensively analyzed a vast amount of microarray data involving CAD from online databases to laboratory experiments. Their results showed that many peripheral immune genes of CAD patients had significant changes compared with the control groups, including but not limited to CXCL8, TNF, SOCS3, TNFAIP3, CD86, C1QB, CD53, C1QC, NCF2, ITGAM, MAPK1, MAPK3, MAPK13, MAPK14, JUN, CHUK, PIK3CB, TLR4, IFNAR1, TLR2, MYD88, IRAK4, CSF3, IL-1A, CCR7 and IL-18 [7–10]. However, these projects' current sample scale and scope are still limited. Besides, few of the screened novel targets got effectively validation or further mechanism exploration, which dramatically restrained their translation to the clinic. Therefore, although we have mastered some convincible evidence, the peripheral immune characteristics and mechanism of CAD still need to be further uncovered.

In this study, we constructed a novel monitoring diagnostic model of advanced-stage CAD with the screened key differentially expressed immune-related genes (DE-IRGs) in the database. We used low-density lipoprotein receptor deficient (LDLR^−/−^) mice (a typical CAD animal model) to determine the correlation between the candidate genes and CAD. Finally, we found that IL13RA1 probably participated in various pathological processes of CAD, which showed a strong potential to be a diagnostic and therapeutic target.

## Materials and methods

### Data preparation

**Fig. 1:**
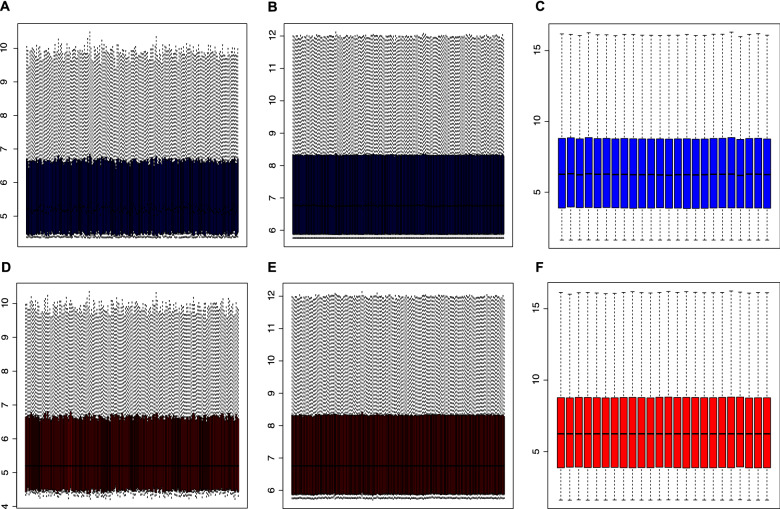
Normalization of original data. Box plots for the expression levels of mRNAs in CAD patients and controls before (**A**–**C**) and after (**D**–**F**) normalization by using the ComBat algorithm. A/D, B/E and C/F are from GSE20680, GSE20681 and GSE42148, respectively. After the operation, the original data was homogenized

The mRNA expression data in the peripheral blood were derived from the Gene Expression Omnibus (GEO; https://www.ncbi.nlm.nih.gov/geo/query/acc.cgi) based on the criteria below: organism (homo sapiens), experiment type (expression profiling by array), disease (CAD). In this study, we eventually obtained three datasets (see corresponding clinical and demography information in Additional file 1: Table S1). The GSE20680 dataset involved 87 CAD patients (more than 1 large artery stenosis ≥ 70% or 2 artery stenosis ≥ 50%) and 108 controls(artery stenosis ≤ 25%, or stenosis > 25%, but < 50%) on the platform of GPL4133 [11]. The GSE20681 dataset included 99 CAD patients (more than 1 main blood vessel ≥ 50% stenosis) and 99 controls(stenosis < 50%) on the platform of GPL4133 [12]. The GSE42148 was composed of 13 CAD patients (diagnosed as CAD patient by Quantitative Coronary Angiography, QCA) and 11 controls (with normal ECG and no clinical symptom) based on the platform of GPL13607[13]. All the data from the three datasets were re-normalized by ComBat algorithm (Fig. 1) to remove the batch effect, and evaluated by principal component analysis (PCA). All the CAD group patients received QCA evaluation, and satisfied at least 1 main blood vessel stenosis≥50%; relative healthy control group was in accordance with all the vessels stenosis < 50% or without ECG change and clinical symptom.

All the CAD patients and controls from the three datasets were randomly but evenly divided into the training set and test set (1:1), respectively. The two sets have the same number and proportion of CAD patients and relatively healthy people.

### Identification of differentially expressed genes (DEGs)

Based on the combined datasets, we used the LIMMA package to screen DEGs between CAD and control groups with the cutoff of |fold change (FC)|> 0.2 and adjusted p < 0.05. Then we visualized them by volcano and heat maps [14].

### Identification of key immune-related genes (IRGs)

We obtained IRGs from Immport database (ImmpDb; https://www.immport.org/). The IRGs (n = 1793) found in the datasets were overlapped with the DEGs (n = 762), and the overlapped part was regarded as CAD progression immune-related genes for the subsequent analysis.

### Protein–protein interaction (PPI) network construction, gene ontology (GO) analysis and KEGG pathway enrichment analysis

The interactions between specified proteins were analyzed via the STRING online tool (http://string-db.org/) [15]. To confirm the effectiveness of this interaction, we limited confidence (combined score) > 0.7. To further explore the underlying biological pathways associated with the identified DEGs, we performed the GO analysis and KEGG pathway enrichment analysis using ClusterProfiler package [16].

### Immune cell subgroups analysis

To better recognize the immune cell characteristics in peripheral blood of relative healthy people and CAD patients, we compared the differences of immune cell subsets in the samples. We used the single sample gene set enrichment analysis (ssGSEA) to compare the differential composition of 28 immune cells between the two groups [17]. The Pearson correlation analysis was used to reveal the correlation between the distribution of immune cells and the expression of the DE-IRGs.

### Establishment and assessment of the immune-based diagnostic model

By feature selection, we reduced the dimension through the least absolute shrinkage and selection operator (LASSO) regression algorithms [18] and determined 14 IRGs to construct the diagnostic monitoring model of CAD. Then we conducted receiver operating characteristic (ROC) curves of the 14 biomarkers based on the training set and test set, and measure the area under the curve to evaluate the diagnostic capacity (area under curve, AUC) of different genes by R software. Similarly, we evaluated the diagnostic power of the combined 14 DE-IRGs by ROC analysis in the training set and test set, respectively.

### Cell lines culture

RAW264.7 cells were purchased from the cell bank of typical culture preservation Committee of Chinese Academy of Sciences (Shanghai, China) and cultivated in high-glucose Dulbecco’s modified Eagle’s medium (DMEM, Cytiva, HyClone Laboratories, United states) with 10% fetal bovine serum (FBS, Procell Life Science & Technology Co., Ltd., China) and 100 U/ml penicillin/streptomycin at 37 ℃ and 5% CO_2_.

### Bone marrow-derived macrophages (BMDMs) harvest

Femur and tibia of C57/B6J(6-week old, male) were flushed with 1 mL of Modified Eagle’s medium(α-MEM, Gbico, United states) with 10% FBS and 100 U/ml penicillin/streptomycin to obtain bone marrow cells. After standing for one night, the medium was transferred to a new 15-mL centrifuge tube. After centrifugation (650 g for 5 min), 2 mL ACK lysis buffer (Biosharp, China) was added to re-suspense cell stack and remove red blood cells (37 °C for 2 min), followed by centrifugation (650 g for 5 min) with another 10 mL PBS solution. The cells were suspended by 1 mL α-MEM with 25 ng/ml recombined murine macrophage colony-stimulating factor(M-CSF, Beyotime biotechnology, China)to induce the maturation and adhesion of macrophages. These cells were then cultured in 6-well plates at 37 °C and 7% CO_2_, until 80% confluence was reached.

### CAD mouse model construction

Eight 7-week-old male LDLR^−/−^ and C57/B6J wild-type mice (Gempharmacy Co., Ltd, China) were raised in the specific pathogen-free (SPF) barrier system of the experimental animal center of Longhua hospital affiliated to Shanghai university of traditional Chinese medicine. After randomization, the CAD model group (LDLR^−/−^ mice) was fed with high fat diet (78.85% chow diet, 21% lard and 0.15% cholesterol) for 20 weeks constantly, and the control group with chow diet. At the end of the 20th week, after anesthesia, the blood from the abdominal aorta was taken to detect lipid metabolism indexes and real-time PCR analysis. Meanwhile, the aortic arch samples were gathered and fixed in 4% paraformaldehyde (PFA) for at least 24 h.

### Transfection

After the cells in 6-well plates achieved 60% confluence, the transfection mixture of Opti-MEM(Gbico, the United states), siRNA (Genomeditech Co., Ltd, China) and lipofectamine2000 (Invitrogen, the United states)which had been rested at room temperature for 20 min, was added to each well (300μL) and incubated at 37 °C and 5% CO_2_ for 6 h. After that, the transfection mixture medium was removed and a new complete medium (2 mL) was added to the wells. The downstream experiments were conducted 48 h after the above process.

### Oil red O staining

RAW264.7 cells (1 × 10^5^) were planked in a 24-well plate and treated with oxidized low-density lipoprotein(ox-LDL, Yeasen company, China)/PBS(100 μg/mL) for 24 h. All the wells were stained with 0.5% Oil Red O-Isopropanol solution (diluted with ddH_2_O in a ratio of 3:2) for 2 h at 37 °C and then were differentiated with 75% ethanol solution for 1 min (3 times). After 3 times washes with PBS, hematoxylin was used to stain nuclei for 2 min. An inverted microscope (Nikon eclipse-e, Japan) was used to capture the representative images.

### RT-qPCR

Following the manufacturer's instructions, we used the EZ-press RNA purification kit (EZBioscience Co., Ltd., China) to extract total RNA from aortas, BMDM and RAW264.7 cells with PBS/ox-LDL intervention. Then the reverse transcription process was carried out with Prime Script™ RT Reagent Kit (Takara, Japan). RT-qPCR reaction was conducted in real-time PCR system (7500, Applied Biosystems) with TB Green Premix Ex Taq Kit (Takara, Japan) including TB green 10 μL, forward primer 0.5 μL, reverse primer 0.5 μL, ROX II 0.4 μL, ddH2O 4 μL and 4.6 μL cDNA samples. The primer sequence (see Table 1) was downloaded from Primer bank (https://pga.mgh.harvard.edu/primerbank/index.html) and synthesized by Shanghai Sangon Biotechnology (Shanghai, China).

**Table 1:** List of primers for real-time PCR analysis

Gene	Oligonucleotide sequence	
CCR9	Forward	5′-CTGGTATTGCACAAGAGTGAAGA-3’
	Reverse	5′-CCACACTGATGCACATGATGA-3’
CSF2	Forward	5′-TCTGAGGTGGATTGGTGTGAG-3′
	Reverse	5′-TGAGGGGTCCAAAGATGAGGA-3′
CXCL2	Forward	5′-CCAACCACCAGGCTACAGG-3′
	Reverse	5′-GCGTCACACTCAAGCTCTG-3′
IL13RA1	Forward	5′-ATGCTGGGAAAATTAGGCCATC-3′
	Reverse	5′-ATTCTGGCATTTGTCCTCTTCAA-3′
IL1A	Forward	5′-CGAAGACTACAGTTCTGCCATT-3′
	Reverse	5′-GACGTTTCAGAGGTTCTCAGAG-3′
INSF5	Forward	5′-CCCCACTCTTGCTCTGTTTCT-3′
	Reverse	5′-GGAAATGCCCCTCCAGATGTC-3′
MSR1	Forward	5′-GCACAATCTGTGATGATCGCT-3′
	Reverse	5′-CCCAGCATCTTCTGAATGTGAA-3′
NR4A2	Forward	5′-GTGTTCAGGCGCAGTATGG-3′
	Reverse	5′-TGGCAGTAATTTCAGTGTTGGT-3′
NTS	Forward	5′-CCCTTTTGCTTTTGAAGCTATGC-3′
	Reverse	5′-GGAGCACAAAGTGCCATCCT-3′
TNFRSF19	Forward	5′-TTCTGTGGGGGACACGATG-3′
	Reverse	5′-AGAAAATTCAGCGCAGATGGAA-3′
ACTIN	Forward	5′-ACTGTCGAGTCGCGTCC-3′
	Reverse	5′-CCCACGATGGAGGGGAATAC-3′
IR13RA1	Forward	5′-ATGCTGGGAAAATTAGGCCATC-3′
	Reverse	5′-ATTCTGGCATTTGTCCTCTTCAA-3′
SCARB1	Forward	5′-GCGCTCGGCGTTGTCA-3′
	Reverse	5′-TGACCTTTTGTCTGAACTCCCTGTA-3′
CD36	Forward	5′-ATGGGCTGTGATCGGAACTG-3′
	Reverse	5′-GTCTTCCCAATAAGCATGTCTCC-3′

### Western blot

Primary BMDMs and RAW264.7 cells were prepared as described above. BMDMs and transfected RAW264.7 cells were scraped from the plates after 24-h induced by 100 μg/mL ox-LDL/PBS. Liquid nitrogen-frozen aortas were grounded into a fine powder. RIPA lysis buffer (with protease inhibitor PMSF) was used to extracted total protein of each sample. BCA method was conducted to determine the concentration of the specimens and quantified with 5 × loading buffer and ddH_2_O. Protein samples were added into 9% SDS-page, electrophoresed at 60 V (Bio-Rad, United states) to be separated for 30 min and then 1.5 h at 90 V. Wet method was performed to transfer protein bands onto the polyvinylidene difluoride membrane (PVDF membrane, Millipore, United states). After blocked in 5% BSA-PBST solution for 1 h, the membrane was incubated with corresponding primary antibodies (IL-6,CST#12,912,1:1000;JAK1,Abcam#ab133666,1:1000;p-JAK1,Abcam#ab138005,1:1000;STAT3,CST#9139,1:1000;p-STAT3,CST#9145,1:1000;VEGF-C,Santa cruz#sc374628,1:200;TGF-β,CST#3711,1:1000;α-SMA, Abcam#ab5694, 1:1000;α-TUBULIN,Abcam#ab7291,1:1000) at 4 °C overnight. TBST was used to wash the blots(6 times × 6 min)before the incubation with proper secondary antibodies(goat anti-rabbit IgG + HRP, Absin#abs20040s, :5000; goat anti-mouse IgG H&L, Yeasen#33201ES60,1:5000) for 1 h at room temperature. Finally, the membrane was washed by TBST again and visualized by ChemiScope 6000 imaging system (CLINX, China) with ECL.

### Statistical analysis

We conducted ROC curves based on the expression features of the DE-IRGs in peripheral blood. Undetected data of PCR test were excluded. Besides, ImageJ software was used to quantify the degree of the lesion. Finally, we used GraphPad Prism 8 via unpaired two-tailed student’s t-test to compare the data of the two groups. All the experiments were independently repeated at least 3 times.

## Results

### Identification of differential mRNA expression in peripheral blood

**Fig. 2:**
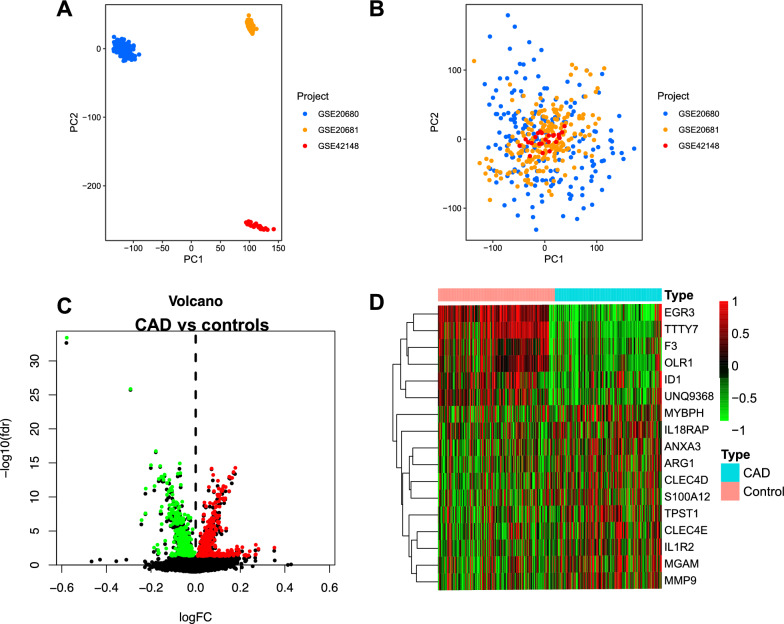
Identification of DEGs. **A**. PCA cluster plot of GSE20680, GSE20681 and GSE42148 before batch effect removal and correction. **B**. PCA cluster plot showed that batch effect has been removed. **C**. Volcano plot of DEGs between CAD and controls. **D**. Heatmap for the top 17 DEGs between CAD and healthy samples. Red: Up-regulation; Green: Down-regulation

The peripheral blood transcriptome of 199 CAD patients and 218 controls was involved in this study. PCA analysis revealed that the batch effect was efficiently removed across the three gene sets (Fig. 2A, B). A total of 762 DEGs were identified, of which 396 genes were up-regulated, while 366 genes were down-regulated (Fig. 2C). The top five up-regulated genes were C17orf78, TTTY21, TMEM213, COL21A1, and SNX31, while the top five down-regulated genes included EGR3, TTTY7, TMEM196, DNAJC28, and OLR1. The heat map showed the genes with |logFC|> 0.2 between CAD patients and controls, suggesting they were likely to participate in the pathological process of CAD (Fig. 2D).

### Functional enrichment analysis for DEGs

**Fig. 3:**
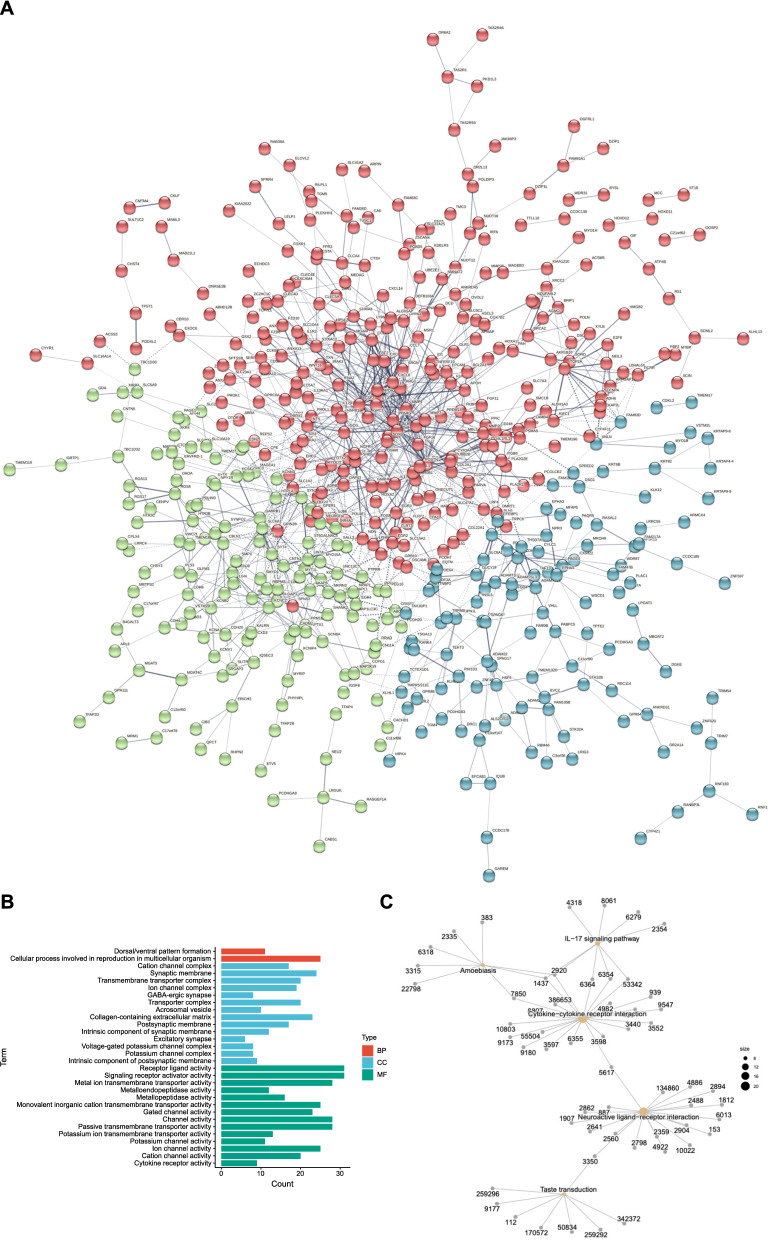
Function enrichment analysis of screened differential genes. **A**. Protein–Protein Interaction (PPI) network construction to reveal the interaction on protein level between different genes. **B**. Gene Ontology (GO) analysis bar plot showed the enrichment of the DEGs in BP, CC, and MF processes. **C**. KEGG-gene analysis plot displays that the DEGs focus on five immune-related signal pathways, and the most is on the cytokine-cytokine receptor interaction (Size = 20)

To explore the relationship between these DEGs and the biological functions involved in the regulation process, we constructed protein–protein interaction (PPI) network. PPI analysis revealed that these genes have strong connections at the protein level (Fig. 3A). GO analysis revealed the profile of the DEGs involved in molecular function, biological process, and cellular component, respectively, such as receptor-ligand activity, transporter complex and cellular process (Fig. 3B). The KEGG pathway enrichment plot showed that the main enriched pathways were cytokine-cytokine receptor interaction, neuroactive ligand-receptor interaction, and IL-17 signaling pathway, indicating a strong correlation with the functions related to inflammation response and neural activity (Fig. 3C).

### DE-IRGs determination and immune-related cell landscape analysis

**Fig. 4:**
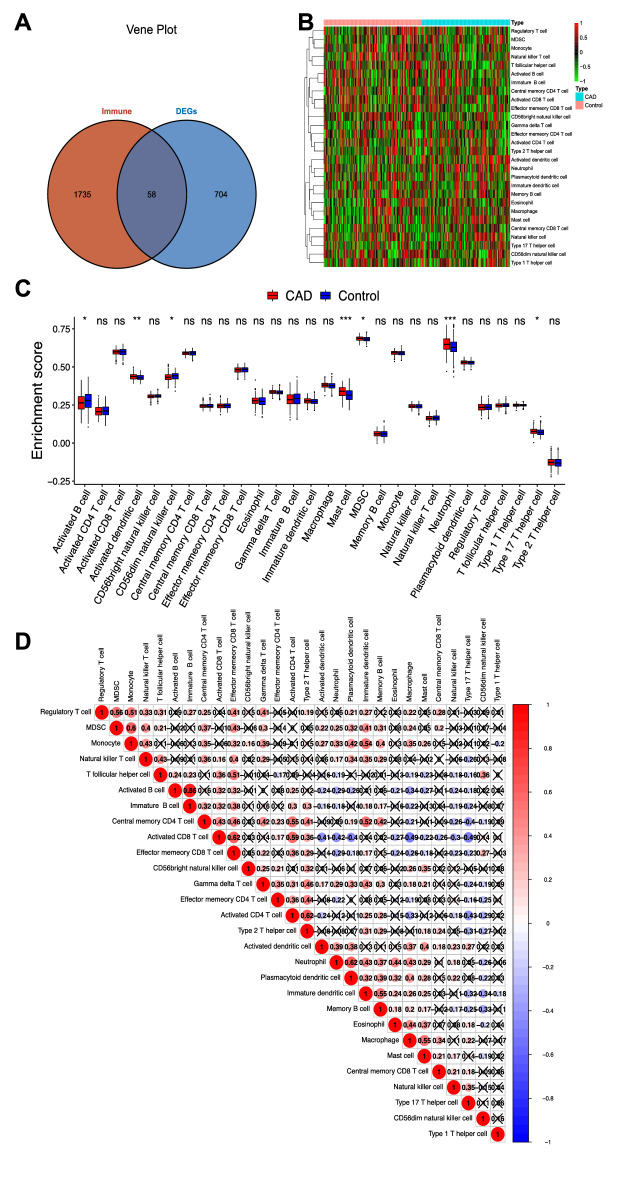
Immune related genes and corresponding subgroups of immune cells. **A**. Venn diagram of 58 DE-IRGs in CAD. **B**. Heatmap to show the main 28 types of immune cells changes in CAD. Red: Up-regulation; Green: Down-regulation. **C**. Box diagram for the enrichment score differences of the immune cells above between CAD group (Red) and control group (Blue). **D**. Correlation bubble chart of 28 types of immune cells. The size of the colored bubbles represents the strength of correlation. Red: Positive correlation; Blue: Negative correlation. The bigger and darker the bubble is, the stronger the correlation is

The above DEGs in CAD patients were overlapped with the IRGs. 58 DE-IRGs in CAD patients were determined (Fig. 4A). Meanwhile, the proportion of seven kinds of immune cell subsets in these chips was found different from CAD to control group. Among them, the proportion of activated dendritic cells, mast cells, neutrophils, Th17 cells and MDSC in CAD increased*(P* < *0.05)*, while the proportion of activated B cells and CD56 killer cells decreased (*P* < *0.05*)(Fig. 4B, C). Additionally, there were correlations between the changed peripheral immune cells (Fig. 4D), such as activated CD4^+^ T cell/Th2 cell(r = 0.62), macrophage/mast cell(r = 0.55), and neutrophil/plasmacytoid dendritic cell(r = 0.62).

### Identification of 14 key peripheral DE-IRGs of CAD and construction of an immune diagnostic model

**Fig. 5:**
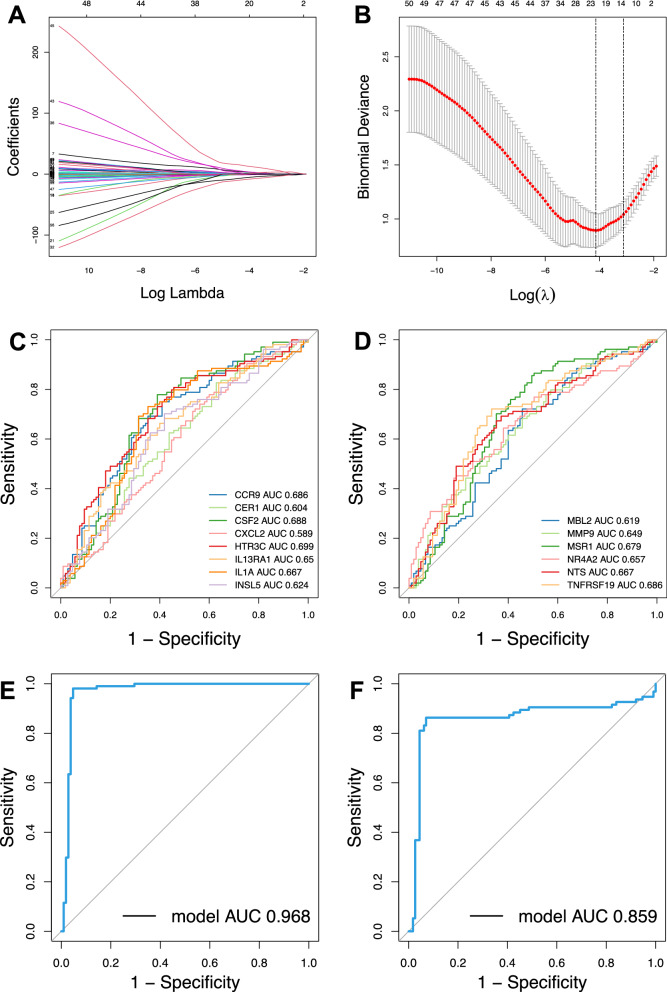
Establishment and test of the 14-gene diagnosis model. **A**, **B**. 14 circulating DE-IRGs were identified as diagnostic biomarkers by LASSO method. **C**, **D**. ROC curves evaluated the diagnostic effect of the 14 genes: CCR9 (AUC = 0.686), CER1 (AUC = 0.604), CSF2 (AUC = 0.688), CXCL2 (AUC = 0.589), HTR3C (AUC = 0.699), IL13RA1 (AUC = 0.650), IL1A (AUC = 0.667), INSL5 (AUC = 0.624), MBL2 (AUC = 0.619), MMP9 (AUC = 0.649), MSR1(AUC = 0.679), NR4RA2(AUC = 0.657), NTS(AUC = 0.667) and TNFRSF19 (AUC = 0.686). E. ROC curves analysis of training set for the diagnostic model including the 14 genes above (AUC = 0.968). F. ROC curves analysis of test set for the diagnostic model including the 14 genes above (AUC = 0.859)

We reduced the dimensions through LASSO regression and eventually determined 14 genes to construct a diagnostic model of CAD (Fig. 5A, B). The diagnostic performance of the identified 14 key peripheral DE-IRGs was evaluated by ROC curves. The areas under ROC curves were CCR9 (AUC = 0.686), CER1 (AUC = 0.604), CSF2 (AUC = 0.688), CXCL2 (AUC = 0.589), HTR3C (AUC = 0.699), IL13RA1 (AUC = 0.650), IL1A (AUC = 0.667), INSL5 (AUC = 0.624), MBL2 (AUC = 0.619), MMP9 (AUC = 0.649), MSR1(AUC = 0.679), NR4RA2(AUC = 0.657), NTS(AUC = 0.667) and TNFRSF19 (AUC = 0.686) (Fig. 5C, D), respectively. However, when all the 14 genes were contained in one diagnostic model, the AUC in the training dataset and test dataset reached 0.968 and 0.859, respectively (Fig. 5E, F). The result proved that these combined DE-IRGs had good potential for diagnosis and might also become promising prevention and treatment targets of CAD.

### Peripheral immune characteristics analysis of CAD patients

**Fig. 6:**
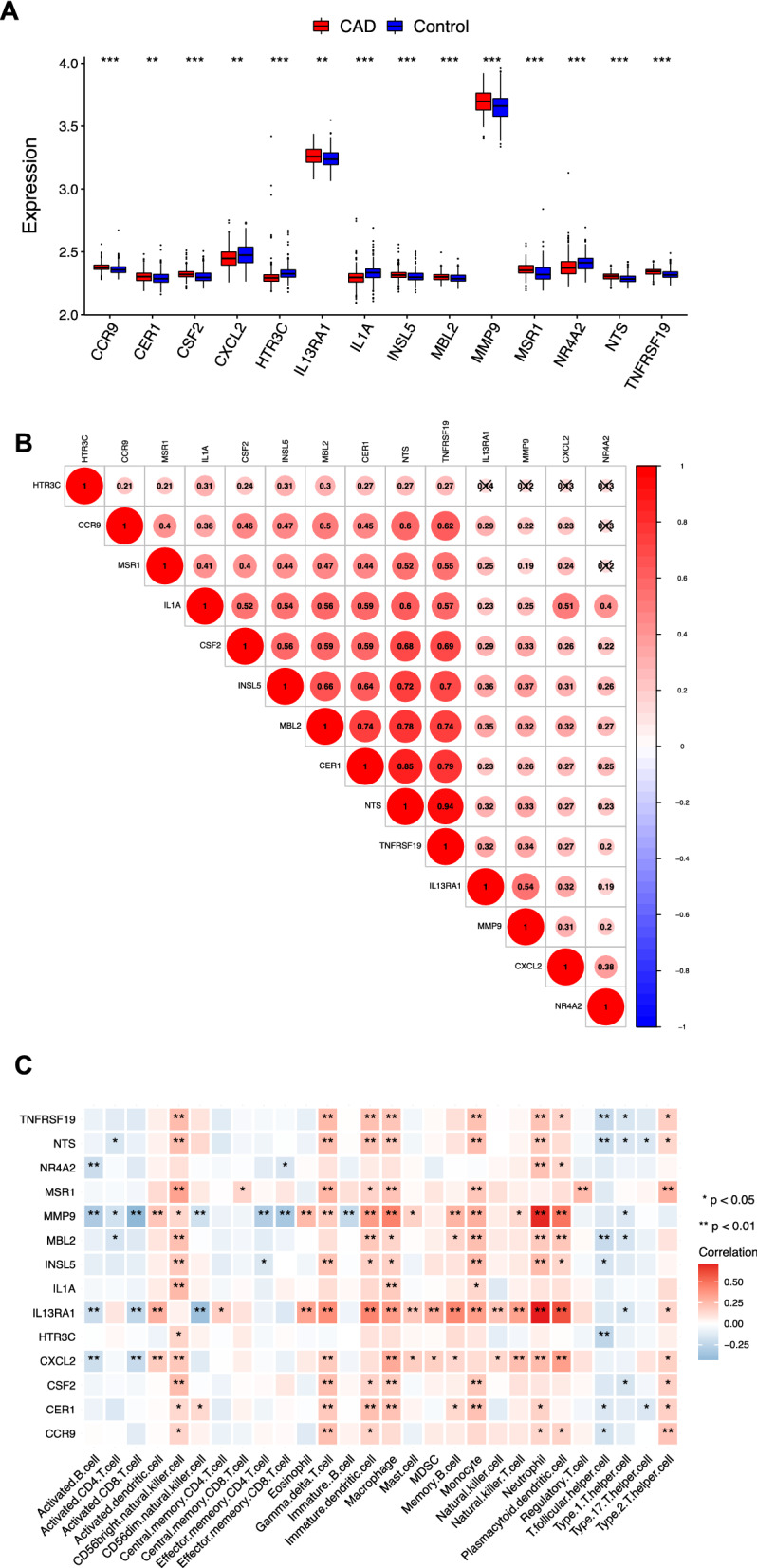
Immune characteristics of peripheral blood in patients with CAD. **A**. The boxplot of the identified mRNAs significantly differed in peripheral blood. **B**. The bubble plot illustrated that most of the identified genes had positive correlations between each other. **C**. Heatmap showed the correlations between the 14 identified genes and 28 types of immune cells. Red: Up-regulation; Blue: Down-regulation. The depth of the colors represented the strength of the correlation. ^*^P value < 0.05, ^**^P value < 0.01, ^***^P value < 0.001

14 key peripheral DE-IRGs showed significant change between CAD and control groups. Among them, CCR9, CER1, CSF2, IL13RA1, INSL5, MBL2, MMP9, MSR1, NTS and TNFRSF19 genes were up-regulation expressed(*P* < *0.05*), while CXCL2, HTR3C, IL1A, and NR4A2 genes were down-regulation expressed (*P* < *0.05*)(Fig. 6A). These genes showed a significant positive correlation, such as CER1/NTS(r = 0.85), CER1/IL13RA1(r = 0.79) and NTS/TNFRSF19(r = 0.94) (Fig. 6B). Considering the important role of multiple immune components in CAD diagnosis and pathological mechanism, we analyzed the interrelation between immune cells and DE-IRGs expression in CAD. For example, IL13RA1 showed a strong up-regulated expression in neutrophil, 3 types of dendritic cell(activated, immature and plasmacytoid), macrophage, memory B cell, monocyte, natural killer cell, MDSC, natural killer cell, mast cell, 2 types of T cell(natural killer and gamma delta) and eosinophil cell (*P* < *0.01*), and rapid down-regulated expression in activated B cell, activated CD8 T cell and CD56dim natural killer cell (*P* < *0.01*). MMP9 showed increased expression in neutrophil, 3 types of dendritic cell(activated, plasmacytoid, immature), monocyte, memory B cell, macrophage, gamma delta T cell, eosinophil (*P* < *0.01*), and descend expression tendency in activated B cell, activated CD8 T, effector memory CD4/CD8 T cell, immature B cell (*P* < *0.01*)(Fig. 6C).

### Verifying the correlation between 10 candidate DE-IRGs and CAD in vivo

**Fig. 7:**
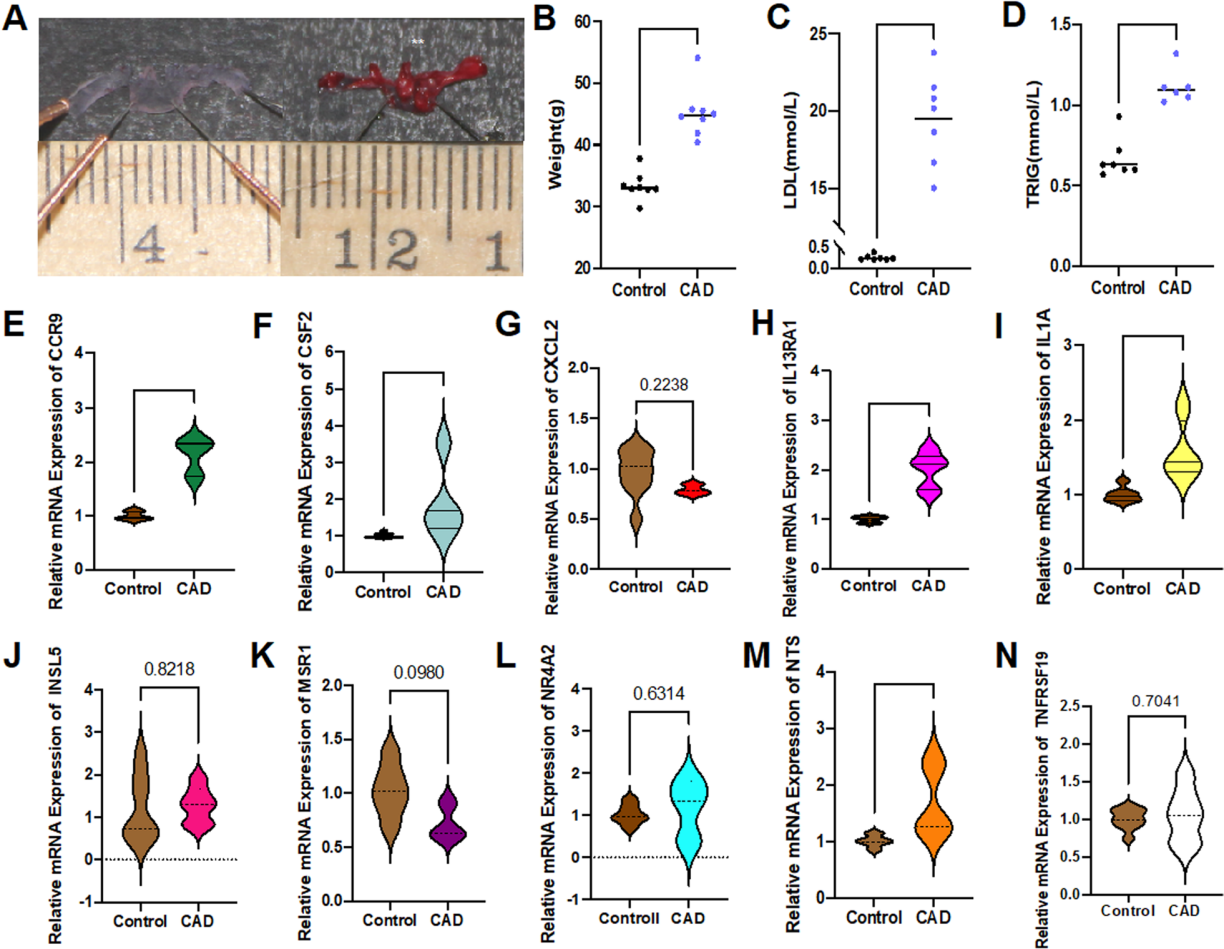
Animal subject validation. **A**. Oil red O staining of mouse aortic arch. **B**. Oil red O staining of mouse aortic arch and corresponding statistical analysis(*P* < *0.01*). **C**–**E**. Weight, LDL and TRIG of CAD mice are far higher than the control group (*P* < *0.0001*). **F**–**O**. Relative mRNA expression value of CCR9^↑^(*P* < *0.0001*), CSF2^↑^(*P* < *0.05*), CXCL2, IL13RA1^↑^(*P* < *0.0001*), IL1A^↑^(*P* < *0.01*), INSL5, MSR1, NR4A2, NTS^↑^(*P* < *0.01*), TNFSF19 detected by real-time PCR. *P < 0.05, **P < 0.01, ****P < *0.0001*. “**”in red represented the PCR result is opposite to the result obtained from the database. “^↑^” represented “upregulation”

At the end of the 20th week, pathological staining (Oil Red O) of mouse aorta arch showed that a large number of lipid plaques gathered, resulting in lumen stenosis (Fig. 7A). The average weight of the CAD model group increased sharply than control group (*P* < *0.0001*) (Fig. 7B). Meanwhile, the peripheral circulating low-density lipoprotein(LDL) and triglyceride (TRIG) levels in the CAD group are high increased (*P* < *0.0001*) (Fig. 7C, D). The detection of whole blood total mRNAs showed that CCR9, CSF2, IL13RA1 and NTS expression in the CAD model group were significantly higher than those in the control group (Fig. 7E–H, M) (*P* < *0.05*), which demonstrated the same trend as those online screened results from the datasets. IL1A, has also been raised in the model group(*P* < *0.01*), showing an opposite tendency to the results of the online data (Fig. 7I). However, no difference between the two groups was found in CXCL2, INSL5, MSR1, NR4A2, and TNFRSF19(*P* > *0.05*)(Fig. 7G, J–L, N).

### IL13RA1 activating JAK1/STAT3 pathway and regulating the function of macrophage in CAD

**Fig. 8:**
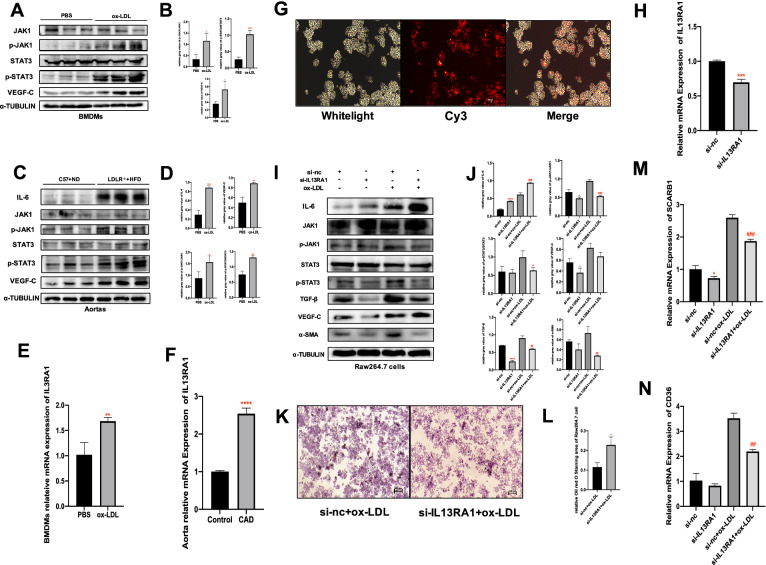
Effect of IL13RA1 knockdown on the expression of CAD-relevant factors. **A**. Significant difference in protein expression between BMDMs treated by PBS or ox-LDL. **B**. Relative statistical results of the protein bands in A(*P* < *0.05)*. **C**. Difference in protein expression between CAD model and negative control group mice aortas. **D**. Relative statistical results of the protein bands in C. **E**. Relative mRNA expression of IL13RA1 in BMDMs treated by PBS or ox-LDL. **F**. Relative mRNA expression of IL13RA1 in CAD model/negative-control group mice aortas. **G**. Reddish fluorescence indicating the transfection efficiency of Cy3-labeled scrambled siRNA. **H**. mRNA expression of IL13RA1 in RAW264.7 after transfected with siRNA. **I**. Difference in proteins expression among si-negative control(si-nc), si-IL13RA1(si-IL13RA1), si-nc with ox-LDL (si-nc + ox-LDL) and si-IL13RA1 with ox-LDL (si-IL13RA1 + ox-LDL) groups. **J**. Relative statistical results of the protein bands in I. **K**. Oil red O staining of ox-LDL-stimulated RAW264.7 cells with/without IL13RA1 knockdown. **L**. Relative statistical results of the stained area in K. **M**. Relative mRNA expression of SCRAB1 among si-nc, si-IL13RA1, si-nc + ox-LDL and si-IL13RA1 + ox-LDL groups. **N**. Relative mRNA expression of CD36 among si-nc, si-IL13RA1, si-nc + ox-LDL and si-IL13RA1 + ox-LDL groups. Si-nc vs. si-IL13RA1, *P < 0.05, **P < 0.01, ****P < *0.0001*. Si-nc + ox-LDL vs. si-IL13RA1 + ox-LDL, *#P* < *0.05, ##P* < *0.01, ###P* < *0.0001*

In vitro, ox-LDL up-regulated the IL13RA1 mRNA expression(*P* < *0.01*), activated JAK1/STAT3 signaling pathway(*P* < *0.05, P* < *0.01*) and also increased the production of VEGF-C in BMDMs(*P* < *0.01*) (Fig. 8A, B–E). In vivo, similarly, in the aortas of 20-week-HFD-diet-induced LDL^−/−^ mice(CAD mouse model), IL13RA1 mRNA expression, phosphate-JAK1, phosphate-STAT3, VEGF-C and IL-6 protein level increased(*P* < *0.01, P* < *0.001)*(Fig. 8C, D–F). After transfecting siRNA into RAW264.7 cells (Fig. 8G), IL13RA1 mRNA expression was significantly knocked down(*P* < *0.001)* (Fig. 8H), which resulted in downregulation of the phosphorylation levels of JAK1 and STAT3, VEGFC, TGF-β and α-SMA(*P* < *0.05, P* < *0.01*)(Fig. 8I, J). The IL13RA1 knockdown groups also had decreased SCARB1 and ox-LDL-stimulated CD36 mRNA levels(*P* < *0.001, P* < *0.01*)(Fig. 8M, N). Interestingly, the knockdown of IL13RA1 increased the IL-6 production(*P* < *0.0001)*(Fig. 8I, J). Under the stimulation of ox-LDL, the above changing trend was consistent and more obvious. Oil red O staining results revealed that the lipid droplets absorbed by macrophages in the IL13RA1 knockdown group decreased (P < *0.05*) (Fig. 8K, L).

## Discussion

Coronary atherosclerosis is a complex pathological process that begins at an early stage of human life. At first, it may be due to some minor vascular abnormalities. Over time, it develops and deteriorates over several years to decades and finally reaches a life-threatening level. In this long process, many pathophysiological factors affect the outcome of the disease. For over one century, it has been believed that circulating lipid disorder was the key cause inducing atherosclerosis-based cardiovascular disease. 20 years ago, Professor Peter Libby put forward the view that inflammation participated in atherosclerosis, which triggered a heated discussion on immune-mediated CAD and was confirmed by several subsequent studies [19]. Today, no one doubts that the immune system impacts the entire process of CAD [20, 21]. In this context, an important method for CAD immunology research is to continuously screen out new differential diagnostic targets through omics and bioinformatics research, subsequently explore mechanisms through animal experiments, or verify reliability in large clinical trials.

Following this line, we obtained high-throughput mRNA data from 199 CAD patients and 218 relative healthy people samples online, then screened 14 DE-IRGs in the peripheral blood from CAD patients to establish a CAD diagnosis model. Among them, CCR9, CER1, CSF2, IL13RA1, INSL5, MBL2, MMP9, MSR1, NTS and TNFRSF19 were highly expressed, while CXCL2, HTR3C, IL1A and NR4A2 gene were low. CCR9, CSF2, IL13RA1 and NTS were validated by the RT-qPCR test and IL13RA1 was further discovered that affect the CAD progression by regulating ox-LDL-stimulated macrophage function.

GO enrichment analysis showed that the differential genes were mainly concentrated in cell proliferation, intercellular communication and mutual regulation, cell adhesion and migration (Fig. 3B). KEGG pathway enrichment analysis suggested that cytokine-cytokine receptor interaction, neuroactive ligand-receptor interaction and IL-17 signaling pathway were significantly enriched (Fig. 3C). Notably, the enrichment of cytokine-cytokine receptor interaction and IL-17 signaling pathway in CAD has been reported in another independent research before [8]. Our results reaffirmed that these two pathways had a strong connection to CAD, revealing that continuous attention should be taken. Moreover, the enrichment associated with neuroactive ligand-receptor interaction indicated simultaneous neurodegenerative changes (such as Alzheimer's disease and Parkinson's syndrome) might occur in the CAD patients involved in this study. The effect of statins or other therapies may also be involved in the above changes [22].

With the development of single-cell sequencing technology [23, 24], the description and recognition for the immune system's participation in the CAD is becoming vivid abundant. Online re-analysing the distribution characteristics of immune cell subsets is therefore revealing enormous value [25]. In atherosclerotic plaque, many circulating immune cells with chemotaxis participate in endothelial injury and lipid infiltration [26]. Our results showed that the number of mast cells, neutrophils, activated dendritic cells, MDSC, and type 17 T helper cells in the CAD group have increased (*P* < *0.05)*. The neutrophil is a crucial cell to promote the progress of atherosclerosis. It can increase the area and instability of plaque by releasing a variety of cytokines and adhesion factors [27], and would also promote macrophage phagocytosis of lipids and MMP-9 level [28, 29]. In our investigation, neutrophils in patients with CAD were strongly associated with the increased expression of IL13RA1 and MMP-9 (*p* < *0.01*). Dendritic cells proliferated and activated in the presence of GM-CSF produced by endothelial cells [30], and might participate in immune signal transmission in the process of CAD. Generally, B1 lymphocytes were considered to have a solid ability to resist atherosclerosis plaque formation and play an essential role in reducing foam cells [31]. In this study, we found the number of activated B cells was reduced compared with those in the healthy group (*P* < *0.05*). Natural killer cell absence resulted in elevated serum cholesterol levels and increased plaque area in mice according to a previous study [32]. In this study, mature killing-capable (CD56dim) natural killer cells of the CAD group showed a downward trend (*P* < *0.*05). However, although differentially expressed in this study, the role of mast cells, MDSC, and type 17 T helper cells in CAD is still not clear enough.

Macrophage scavenger receptor 1(MSR1) and nuclear receptor subfamily 4 group A member 2(NR4A2) have been reported to play a protective role in CAD. MSR1 inhibits TNFs by promoting the expression of IL-10αexpression to regulate immunity, which is beneficial to the stability and regression of AS and myocardial repair after infarction [33]. NR4A2 is a transcription factor to protect cardiomyocytes by transcriptionally inhibiting CCR5 and promoting macrophage polarization to M2 type [34], therefore seemed to play a positive role in anti-inflammation.

Colony-stimulating factor 2(CSF2), C–C motif chemokine receptor 9(CCR9), matrix metallopeptidase 9(MMP9) and neurotensin (NTS) seem to exacerbate the progress of CAD. CSF2 expresses granulocyte colony-stimulating factor (GM-CSF), GM-CSF plays a critical role in promoting the transformation from circulatory monocytes to local macrophages. Recent research has reported that it leads to the susceptibility of macrophages to apoptosis by mediating IL-23 activated oxidation pathway, which results in plaque progression in advanced atherosclerosis [35]. The CCR9 was previously reported to be related to the process of inflammatory expression and myocardial remodeling after myocardial infarction. After the gene was knocked out, it reduced cardiac-related inflammatory factors such as IL-6 and IL-1β, TNF-α and so on, which may be mainly through NF-κB and MAPK signaling pathways to regulate the production of myocardial hypertrophy [36]. MMP9 regulation is closely related to multiple signaling ways of cardiovascular disease, atherosclerotic plaque instability, and myocardial tissue repair after infarction. Previous studies have already shown that the change of MMP9 level in circulation is an independent predictor of atherosclerosis [37]. Circulating NTS is a previously reported risk factor for cardiovascular disease. Its up-regulation increased the risk of type I diabetes and multiple atherosclerotic diseases by promoting lipid absorption [38].

Mannose-binding lectin 2(MBL2), C-X-C motif chemokine ligand 2(CXCL2), interleukin 1α (IL-1A) and TNF receptor superfamily member 19(TNFRSF19) only showed a correlation with CAD. Previous studies have shown that MBL2 is highly expressed in peripheral blood of patients with CAD [39], related to various heart risks, and has led to the critical value of early diagnosis. CXCL2 and IL-1αwere also found up-regulated in the atherosclerotic mouse model [40, 41]. However, CXCL2 had no significant difference in our animal validation, while up-regulation of IL-1A was displayed in the CAD group, which is opposite to the dataset analysis result. The probable causes of the bias might include the different baseline status of involved cases, the immune system gap between humans and mice, and the systematic errors between the two experimental methods. TNFRSF19 is a serum biomarker of chronic inflammation. Previous studies have reported the increased expression of TNFRSF19 in CAD [42], but these studies were very preliminary.

Furthermore, 5-hydroxytryptamine receptor 3C (HTR3C), cerberus 1(CER1) and insulin-like 5(INSL5) were screened out for the 1st time and might have the potential to participate in the CAD process. As far as we know, they have never been reported to be directly related to CAD before, but some have been confirmed to affect the changes of immune targets in circulation. For example, INSL5, as a peptide hormone, after intraperitoneal injection, leads to significant changes in inflammatory factors such as IL-5, IL-7, M-CSF, IL-15 and IL-27 [43], and these inflammatory signals typically play a regulatory role in CAD. However, more direct evidence is needed to explore the causal relationship between them and CAD.

The detection of biomarkers in peripheral blood is simpler and faster than invasive methods, which is suitable for a wide range of community risk screening and timely progression assessment. Based on this, we constructed a peripheral immune diagnosis model with the 14 screened genes, which showed good diagnostic efficiency in the diagnostic tests of the training set (AUC = 0.968) and test set (AUC = 0.859). In addition, four related candidate genes CCR9 (*P* < *0.0001*), CSF2 *(P* < *0.05)*, IL13RA1 (*P* < *0.0001*) and NTS (*P* < *0.01*) (Fig. 7E, F–H–M) were obtained in the in-vivo correlation verification of 10 novel targets. Among them, IL13RA1 shows a strong relevance (*P* < *0.0001*), and belongs to one of the receptors involved in the main enrichment signal pathway cytokine-cytokine receptor interaction (Fig. 3C), which is also significantly related to the distribution of macrophages (Fig. 6C). We explored its role in macrophages in vitro.

Interleukin 13 receptor α1(IL13Rα1), connected with interleukin 4 receiver α1(IL4α1), forms a receptor complex, which is the main receptor of interleukin 13 (IL-13) and interleukin (IL-4). It triggers phosphorylation activation of downstream JAK1, STAT3, and STAT6, and regulates cell proliferation and migration activities. As a main signal of the macrophage alternative activation pathway, IL13RA1 participate in releasing IL-10 and TGF- β to inhibit the inflammatory response and strengthen the phagocytosis and migration of macrophages [44], therefore it is speculated to play a positive role in CAD. However, the direct evidence of IL13RA1 affecting CAD is still limited. Although it is generally believed that M2 macrophages help reduce inflammation in atherosclerosis, thus alleviating the development of CAD, there is also evidence that M2 macrophages formed in the early stage of AS are more likely to ingest cholesterol and form foam cells [45]. Meanwhile, under the induction of typical proinflammatory factors, they are more likely to transform into M1 macrophages than the unpolarized ones [46, 47]. In this study, IL13RA1 mRNA expression in aortic arch samples of HFD-fed LDLR^−/−^ mice was significantly up-regulated. Consistently, under ox-LDL stimulation, IL13RA1 mRNA expression in BMDMs showed the same trend. JAK1/STAT3 signaling pathway was activated both in vivo and in vitro, suggesting that the IL13RA1/JAK1/STAT3 may be involved in the formation of CAD lesions.

We assumed that IL13RA1 played a negative role in CAD. The expression of IL13RA1 in macrophages was interfered with by using small interfering RNA (si-IL13RA1). The results showed that IL13RA1 knockdown macrophages reduced lipid uptake under the stimulation of ox-LDL (P < 0.05). When ox-LDL was not added, no difference in the activation of JAK1/STAT3 between the two groups occurred, but after ox-LDL was added, the phosphorylation ratio of the two groups was significantly up-regulated. The up-regulation range of the knockdown(si-IL13RA1 + ox-LDL) group was significantly lower than that of the negative control(si-nc + ox-LDL) group (*P* < *0.001, P* < *0.05*). Regardless with/without the stimulation of ox-LDL, in the IL13RA1 knockdown group, TGF-β and α- SMA expression both decreased (*P* < *0.01*). However, the decrease of VEGF-C expression only occurred in the IL13RA1 knockdown group without ox-LDL induction. (*P* < *0.05*). These results show that the reduced expression of IL13RA1 leads to the reduced potential of macrophages to resist inflammation and promote fibrosis. Furthermore, regardless with/without ox-LDL induction, the expression of IL-6 was remarkably up-regulated by IL13RA1 knockdown (*P* < *0.0001, P* < *0.001*), suggesting that the deletion of IL13RA1 aggravated the inflammatory response of macrophages. Besides, under the stimulation of ox-LDL, the knockdown of IL13RA1 revealed lower expression of SCARB1 (SR-B1) and CD36 mRNA levels (*P* < *0.001, P* < *0.01*). Since these two kinds of receptors mediate cholesterol efflux and cholesterol uptake in macrophages, respectively, proving that the presence of IL13RA1 enhances the fluidity of cholesterol inside and outside macrophages. However, from the results of our experiments, the deletion of IL13RA1 finally led to more lipid uptake by macrophages.

Thus, IL13RA1 seems to play multiple roles in CAD. On the one hand, it may promote the expression of CD36, activates JAK1/STAT3, strengthens the lipid loading of macrophages, and accelerates the formation of foam cells; On the other hand, it can also reduce the inflammatory response by promoting the TGF-β and inhibiting IL-6, promotes the formation of plaque fibrous cap by increasing α-SMA, and enhances cholesterol efflux by up-regulating SR-B1. In addition, IL13RA1 also promotes the production of VEGF-C, which is believed to accelerate the process of plaque inflammation and lipid accumulation by promoting the formation of pathological lymphatics in the early stage of atherosclerosis [48], and also plays an active role in plaque regression and cardiac function recovery in the later stage [49].

## Conclusion

CCR9, CER1, CSF2, IL13RA1, INSL5, MBL2, MMP9, MSR1, NTS, TNFRSF19, CXCL2, HTR3C, IL1A, and NR4A2 are the differentially expressed immune-related genes of CAD that we screened from online datasets. We also analyzed immune cell infiltration. We constructed a diagnostic model with good efficiency based on the above 14 genes. Therein, CCR9, CSF2, IL13RA1, and NTS were confirmed to be abnormally expressed in HFD-fed LDLR^−/−^ mice. We focused on IL13RA1 and found it participates in regulating the transport of lipids, inflammation, and fibrosis in macrophages, therefore affecting CAD.

One goal of this study is to provide a simple case for translational research on DE-IRGs from CAD samples screened online. However, this study is still preliminary with limitations. Large clinical trials and single-cell level studies will greatly optimize the current work, which is our future direction.

## Supplementary Information

**Additional file 1**: **Table S1** Clinical and demographic characteristics of GSE20680, GSE20681 and GSE42148

## Data Availability

The original contributions presented in the study are included in the Supplementary Materials, further enquiries can be directed to the corresponding author.
