# Supplementary material for: Identification of diagnostic biomarkers and therapeutic targets in peripheral immune landscape from coronary artery disease
Source: J Transl Med. 2022 Sep 5;20:399. doi: 10.1186/s12967-022-03614-1 (PMC9444127; doi:10.1186/s12967-022-03614-1)
Supplement: Supplementary file 1 — Additional file 1: Table S1 Clinical and demographic characteristics of GSE20680, GSE20681 and GSE42148 [file 12967_2022_3614_MOESM1_ESM.pdf]

Table S1 Clinical and demographic characteristics of GSE20680, GSE20681 and GSE42148

		GSE20680			GSE20681			GSE42148(20 samples was provided)*		
	Clinical factors N(%)	Controls N=108	CAD N=87	P	Controls N=99	CAD N=99	P	Controls N=10	CADs N=10	P
General information	Sex (male)	55(50.9%)	58 (66.7%)	0.039	75(75.8%)	75 (75.8%)	0.868	10 （100%）	10 （100%）	> 0.99
	Age (yrs)	55 ± 11	63 ± 10	<0.01	55 ± 12	62 ± 11	<0.01	46.70 ± 1.430	48.7±0.761	0.779
	Caucasian	56 (51.9%)	60 (69%)	0.023	85(85.9%)	92 (92.9%)	0.166	—	—	—
	BMI	32 ± 7	30 ± 6	0.098	30 ± 7	30 ± 6	0.722	23.98±1.603	24.26±0.87	0.878
	Waist circumference (cm)	—	—	—	—	—	—	87.10±4.100	87.90 ±1.99	0.863
	Hip circumference (cm)	—	—	—	—	—	—	88.99±3.620	88.20±1.610	0.863
	Waist/hip ratio (cm)	—	—	—	—	—	—	0.97 ±0.029	0.99±0.016	0.709
Medical history	Current Smoker	41 (38%)	45 (51.7%)	0.075	14(14.1%)	25 (25.3%)	0.074	5 (50.0%)	5 (50.0%)	0.672
	Systolic BP	144 ± 22	153 ± 25	<0.01	132 ± 17	138 ± 18	< 0.01	—	—	—
	Diastolic BP	83 ± 13	87 ± 15	0.077	82 ± 11	80 ± 12	0.271	—	—	—
	Hypertension	67 (62%)	65 (74.7%)	0.084	55(55.6%)	65 (65.7%)	0.191	2 (20.0%)	4 (40.0%)	0.314
	Dyslipidemia	55 (50.9%)	58 (66.7%)	0.039	50(50.5%)	69 (69.7%)	0.009	—	—	—
	Diabetes mellitus	—	—	—	—	—	—	4 (40.0%)	4 (40.0%)	0.675
Laboratory studies	-TC (mg/dL)	—	—	—	—	—	—	154.30±7.949	129.11±8.200	0.052
	-TG (mg/dL)	—	—	—	—	—	—	190.60±27.06	149.89±17.70	0.227
	-HDL-c(mg/dL)	—	—	—	—	—	—	33.70±1.814	37.22 ±1.498	0.072
	-LDL-c(mg/dL)	—	—	—	—	—	—	83.480±9.857	61.91±6.17	0.084
	FBS mg/L	—	—	—	—	—	—	115±21.310	104.00±19.67	0.705
	Neutrophil Count	3.8 ± 1.2	4 ± 1.3	0.392	3.9 ± 1.2	4.3 ± 1.5	0.037	—	—	—
	Lymphocyte Count	1.8 ± 0.7	1.9 ± 0.7	0.87	2 ± 0.7	1.9 ± 0.6	0.239	—	—	—
Treatment	Statin	—	—	—	—	—	—	0	9(90.0%)	< 0.01
	Beta blocker	—	—	—	—	—	—	0	7(70.0%)	< 0.01
	Calcium channel	—	—	—	—	—	—	0	0	> 0.99
	Blocker	—	—	—	—	—	—	1(10.0%)	1(10.0%)	0.901
	ACE inhibitor	—	—	—	—	—	—	1(10.0%)	3(30.0%)	0.053
	Antiplatelet	—	—	—	—	—	—	0	5(50.0%)	< 0.01
	Hypoglycemic agents	—	—	—	—	—	—	4(40.0%)	2(20.0%)	0.239
	Nitrate	—	—	—	—	—	—	0	8(80%)	< 0.01

* Available sample data/total sample data: Controls(N=217/218), CADs(N=196/199)
